# Fruits Produce Branched-Chain Esters Primarily from Newly Synthesized Precursors

**DOI:** 10.1021/acs.jafc.4c10677

**Published:** 2025-02-07

**Authors:** Philip Engelgau, Sumithra K. Wendakoon, Nobuko Sugimoto, Randolph M. Beaudry

**Affiliations:** † Department of Horticulture, Michigan State University, East Lansing, Michigan 48823, United States; ‡ Ryukoku University, Department of Agricultural Sciences, Otsu 520-2194, Japan

**Keywords:** acetohydroxyacid synthase, acetolactate synthase, apple (*Malus* ×*domestica* Borkh.), banana (*Musa* spp.), branched-chain amino acids, branched-chain ester precursors, citramalate synthase, flowering quince (*Chaenomeles* ×*superba*), fruit ripening, sulfonylurea

## Abstract

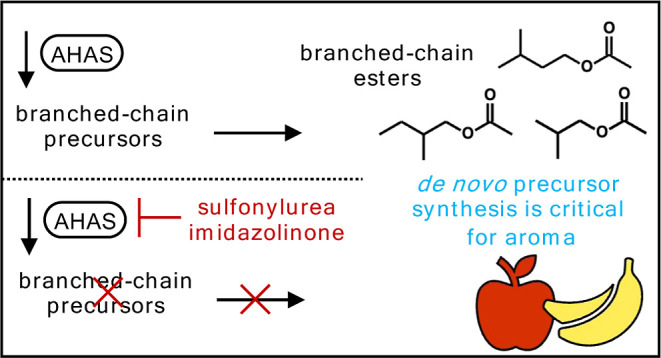

Inhibitors of acetohydroxyacid synthase (also known as acetolactate synthase), the common enzyme of branched-chain amino acid biosynthesis, were used as tools to discern the contribution of newly synthesized precursors (i.e., branched-chain amino acids and α-ketoacids) to branched-chain ester formation in ripening apple (*Malus* ×*domestica* Borkh.), banana (*Musa* spp.), and flowering quince (*Chaenomeles* ×*superba*) fruits. After treatment, *anteiso*- and *iso*-branched-chain esters (i.e., those related to isoleucine, and valine and leucine, respectively) universally decreased in content by at least 90%. Among free amino acids, only the branched-chain amino acids, with correspondingly reduced branched-chain esters, had a lesser concentration following treatment with the inhibitor. Branched-chain ester production recovered after subsequent feeding with precursor compounds. Our results ultimately reject the hypothesis that *anteiso*- and *iso*-branched-chain esters of ripening fruits are primarily derived from preexisting sources and instead support the hypothesis that these esters are largely the product of *de novo* precursor biosynthesis.

## Introduction

**Figure 1:**
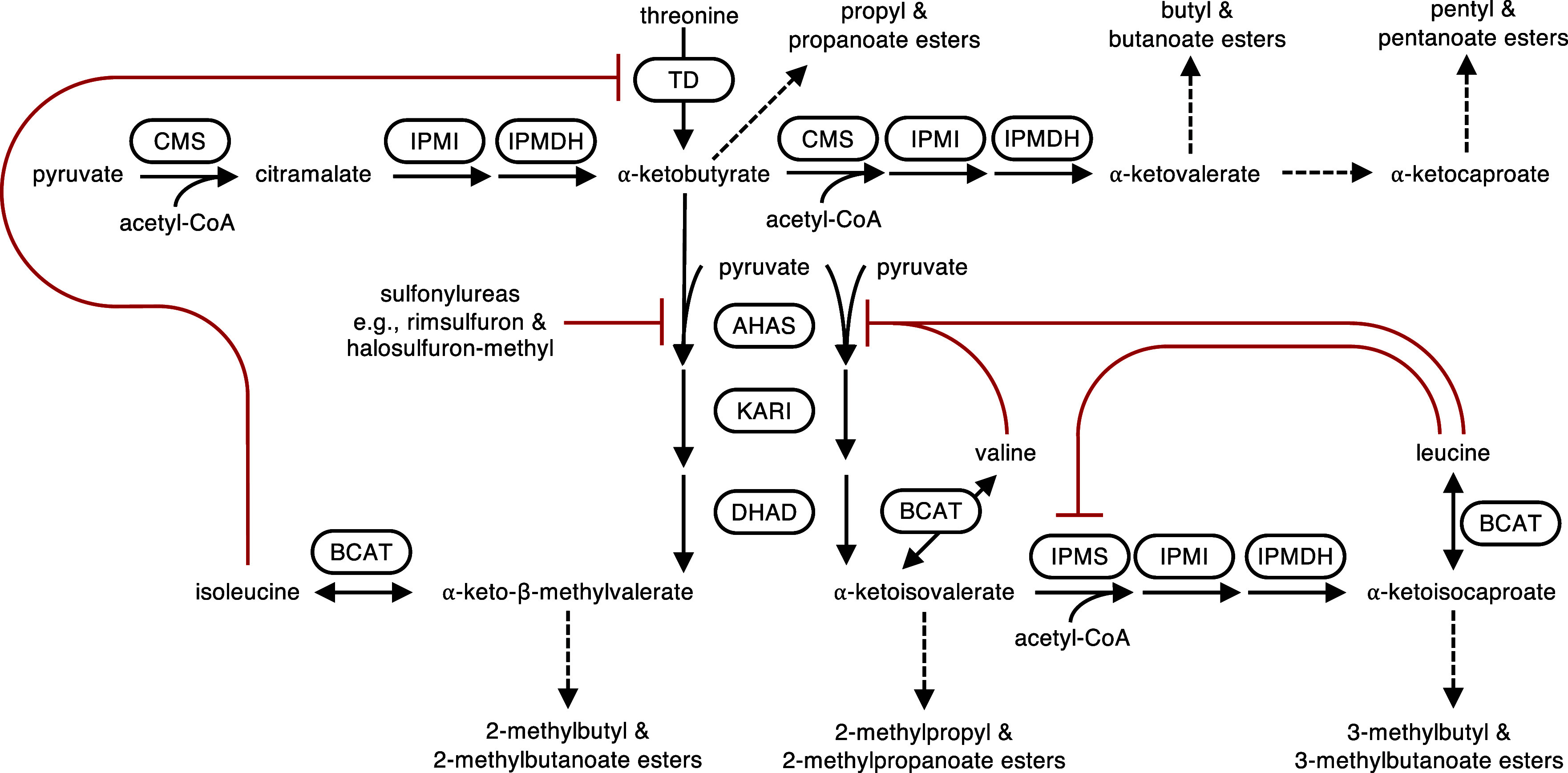
Branched-chain amino acid biosynthesis and the citramalate synthase pathway. Reactions shown as solid arrows; those understood to be freely reversible are depicted with double-ended arrows. Abbreviated pathways shown with dashed arrows. Enzymes shown in curved boxes. Principal inhibitory interactions drawn with red lines. Minor inhibitory mechanisms not shown: antagonism of isoleucine feedback at TD by valine and inhibition of AHAS by isoleucine. Note that of the fruits discussed herein, CMS has only been identified in apples. α-Ketocaproate elongation via CMS has not been observed in apple fruits. AHAS = acetohydroxyacid synthase (also known as acetolactate synthase), BCAT = branched-chain aminotransferase, CMS = citramalate synthase, DHAD = dihydroxyacid dehydratase, IPMDH = isopropylmalate dehydrogenase, IPMI = isopropylmalate isomerase, IPMS = isopropylmalate synthase, KARI = ketol-acid reductoisomerase, TD = threonine deaminase.

For over half a century, a relationship has been known to exist between the branched-chain esters, which act as impact flavor notes for many popular fruits, and branched-chain amino acids. Specifically, feeding studies have demonstrated an interchange of labeled carbons between exogenously fed branched-chain amino acids and emanated branched-chain esters, linking 2-methylbutyl and 2-methylbutanote esters to isoleucine metabolism, 2-methylpropyl and 2-methylpropanoate esters to valine metabolism, and 3-methylbutyl and 3-methylbutanoate esters to leucine metabolism (Figures 1 and S1).^1−3^ While substantial effort has been given to understand the conversion of branched-chain amino acids and their respective α-ketoacids into esters, the means that fruits use to supply said precursors has never been directly investigated. Despite this lack of concrete evidence, it has regularly been stated or implied that branched-chain aroma biosynthesis is sourced by catabolic means—seemingly inferred from the above-mentioned feeding experiments.^4−9^

Ripening in fruits is a dynamic process involving sequentially induced modifications to many metabolic processes.^10^ It would seem inconsistent to suggest that autonomous aroma biosynthesis, the often-terminal feature of ripening and thus the ultimate attractant for consumption and seed dispersal,^11^ is not also an actively regulated and developmentally controlled process. We hypothesize that the entirety of autonomous ester formation is under programmed regulation and thus propose that branched-chain esters are derived from newly synthesized precursors via anabolic processes rather than catabolic processes.

The metabolites that directly link primary and specialized metabolism for branched-chain ester production are the branched-chain α-ketoacids (Figure 1). The quantity of branched-chain α-ketoacids reflects the quantity of the branched-chain amino acids because transamination between the two, facilitated by branched-chain aminotransferases, is freely reversible.^12,13^ While the amino acids are more routinely measured and exogenously applied to fruits than the α-ketoacids, the branched-chain α-ketoacids are the more direct precursors to branched-chain esters.^14,15^ However, as the following research is principally concerned with processes that are upstream from the conversion of α-ketoacids into esters, the branched-chain amino acids and α-ketoacids will collectively be considered as a precursor pool for branched-chain ester biosynthesis herein.

There are several lines of evidence that indirectly support the *de novo* synthesis of branched-chain ester precursors. Among the free amino acids of ripening apple (*Malus* ×*domestica* Borkh.) and banana (*Musa* spp.) fruits, only those with related branched-chain volatiles produced by the fruit undergo a marked increase that is concomitant with aroma emanation (i.e., isoleucine in apple, and valine and leucine in banana).^14,16^ Catabolic processes would not be expected to produce such coincidental results, implying that these fruits are actively engaging the synthetic processes of branched-chain amino acids and α-ketoacids.

Sugimoto et al.^17^ further demonstrated the importance of *de novo* precursor production in apple fruit through the elucidation of citramalate synthase’s role in providing an alternative synthetic route that effectively circumvents isoleucine’s feedback inhibition of threonine deaminase, and, in so doing, produces >80% of the precursor pool for 2-methylbutyl and 2-methylbutanoate ester production (Figure 1). Apple cultivars that lack a catalytically active allele of citramalate synthase produce minimal quantities of the said esters. The role of *de novo* precursor synthesis has likewise recently garnered greater consideration in other fruits as well, such as tomato^18^ and muskmelon.^19^

**Figure 2:**
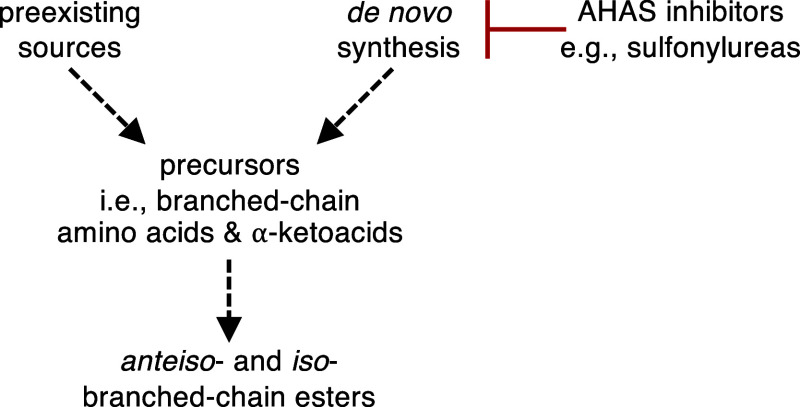
Simplified, hypothetical routes of fruit volatile ester precursor biogenesis. Precursors of *anteiso-* and *iso-*branched-chain esters are potentially supplied by preexisting sources and/or *de novo* synthesis. The latter can be arrested by application of acetohydroxyacid synthase (AHAS) inhibitors, allowing for assessment of precursor pathway contributions.

To further our understanding of the source of ester precursors, we sought to definitively determine whether branched-chain esters in fruit are made from preexisting amino acids and α-ketoacids and/or made from those newly synthesized during ripening (Figure 2). Apple and banana fruits were chosen as ideal testing materials due to (1) the relatively large proportion of branched-chain esters in their aroma profiles, (2) the availability of substantial descriptive biochemical data for the metabolites of interest, and (3) the diversity of adaptations and physiologies among climacteric fruits that they represent. To extend the inquiry to nonhuman-consumed fruits, ornamental flowering quince (*Chaenomeles* ×*superba*), a little explored apple relative, was included.

We hypothesized that if ester production is dependent upon anabolic precursor synthesis in ripening fruits, then targeted inhibition of the canonical biosynthetic pathway for branched-chain amino acids and α-ketoacids should simultaneously prevent the accumulation of said compounds as well as their downstream metabolites, including branched-chain esters. On the other hand, if the branched-chain amino acids and α-ketoacids utilized in aroma biosynthesis are preexisting, such as being derived from protein degradation, then branched-chain ester synthesis should persist or be minimally disrupted by inhibition of *de novo* synthesis (Figure 2).

## Materials and Methods

### Plant Material

‘Gala’, ‘Empire’, ‘Jonagold’, and ‘Red Delicious’ apple fruits (*Malus* ×*domestica* Borkh.) were harvested from local orchards at commercial maturity and transported to the laboratory during the 2022 and 2023 seasons. As determined by previously described methods,^14^ the fruits were at the developmental onset of ripening (Table S1) with no subjectively discernible aroma. ‘Gala’ and ‘Empire’ fruits began treatment (see below) immediately after arrival in the laboratory. ‘Jonagold’ fruits were held in the air at 0 °C for 2 days before transfer to controlled atmosphere (CA) storage (1.5% O_2_, 3% CO_2_, 0 °C) and then kept in CA for 12 days to suppress ethylene action before the initiation of inhibitor treatment. ‘Red Delicious’ fruits were treated with 50 nL·L^–1^ 1-methylcyclopropene (MCP) for 24 h at 0 °C. MCP was evolved from a commercial MCP product (EasyFresh, Fine Americas, Walnut Creek, CA) in sealed chambers 2 days after harvest as previously described.^20^ The fruits were then stored in air at 0 °C for five months and were warmed to 22 °C for 24 h before sulfonylurea treatment. This low-dosage MCP treatment had very mild effects on physiology.^21^ After removal from storage and warming, the respiration rate of MCP-treated fruits was 88.7% that of untreated fruits, and the firmness was 57.2 N (that of untreated fruits was 44 N). Based on the internal ethylene concentration (∼100 μL·L^–1^) and the total ester production (elevated and trending upward), the fruits were considered to be in an advanced stage of ripeness but not senescent. Overall, fruit storage conditions were used as a convenient means for manipulating the ripening stage of experimental material, not as experimental variables.

Banana fruits (*Musa* spp. AAA group, Cavendish subgroup, cv. Valery) that had not been treated with ethylene were obtained from a local supermarket produce distribution and ripening center (Meijer/Chiquita, Lansing, MI) on the day of their arrival. ‘Valery’ fruits were held at 13.5 °C until treatment. It was noted that fresher fruits provided more consistent results compared to older ones, so fruits were typically used within the first 10 days after procurement.

‘Dr. Banks Pink’ flowering quince fruits (*Chaenomeles* ×*superba*) were collected from accession CC7985*05 on the Michigan State University campus grounds. Fruits were subjectively determined to be producing aroma at the time of harvest.

### Treatment and Sample Preparation

#### Apple Fruit at an Early Stage of Ripening and Flowering Quince Fruit

Whole apple fruits, freshly harvested and at an early stage of ripening (“ripening”), were held at room temperature (22 °C) and rubbed daily with 3 mL of a freshly made 1 mM solution of the inhibitor rimsulfuron (*N*-((4,6-dimethoxypyrimidin-2-yl)aminocarbonyl)-3-(ethylsulfonyl)-2-pyridinesulfonamide, made from DuPont Matrix SG, 25% w/w active ingredient; 0.1% Tween 20) or water solution containing 0.1% Tween 20 before preparation with further treatments. The flowering quince fruit was smaller than the apple fruit and had 2 mL of the control or treatment solutions applied daily at 22 °C. ‘Gala’, ‘Empire’, and ‘Jonagold’ apple fruits were treated for four, nine, and seven days before further preparation, respectively. Flowering quince fruits were treated for 6 days before analysis. Headspace volatile emissions were monitored for three or four representative fruits during the incubation/treatment period to determine the appropriate times for further treatments. A time course of the suppression of the dominant branched-chain ester of apple fruit aroma, 2-methylbutyl acetate, can be seen in Figure S2B. During method testing, 1 mM solutions of the imidazolinone imazamox were also assessed with apple fruits (2-[4,5-dihydro-4-methyl-4-(1-methylethyl)-5-oxo-1*H*-imidazole-2-yl]-5-(methoxymethyl)-3-pyridinecarboxylic acid, made from Raptor, 12.1% w/w active ingredient; 0.1% Tween 20).

α-Ketoacids (2-oxoacids) were fed to ‘Empire’ and ‘Jonagold’ fruits by preparing vials of excised peel tissue discs as previously described^17^ in 22 mL glass vials using 20 μL of treatment solution on the prepared paper-peel disc units. The treatment solution contained 20 mM methanol, 0.1% Tween 20, 20 mM α-keto-β-methylvalerate (3-methyl-2-oxopentanoate), α-ketoisovalerate (3-methyl-2-oxobutanoate), or α-ketoisocaproate (4-methyl-2-oxopentanoate), and, when appropriate, 1 mM rimsulfuron.

Acetate feedings were likewise performed with excised peel tissues in 22 mL glass vials and 20 μL of treatment solution. ‘Gala’ fruits were fed with 10 mM methanol, 10 mM acetate (unlabeled or 1,2-^13^C_2_) pH 7 balanced with KOH, 0.05% Tween 20, and 0.5 mM rimsulfuron if appropriate. These concentrations were doubled for ‘Empire’ and ‘Jonagold’ fruits. The expected label patterning discussed in detail, as well as calculations of percent enrichment, can be seen in ref (17).

#### Apple Fruit at a Late Stage of Ripening

‘Red Delicious’ apple fruits held in refrigerated air for 5 months (see above) plus 1 day at 22 °C and at an advanced ripeness stage (“ripe”) were treated with rimsulfuron (1 mM) with 0.2% Tween 20 on day 0 of the study and for the next three consecutive days as described above. The fruits were evaluated daily for volatile emissions (see below) for 8 days. On days 0, 2, 4, and 7, representative treated and untreated fruits were selected for tissue collection for metabolite analysis (see below). On day 7, samples of fruit peel were fed, as described above, with water or 20 mM isoleucine or α-keto-β-methylvalerate.

#### Banana Fruit

Banana fruits at an early stage of ripening were infiltrated with AHAS inhibitors by two means, both inspired by past studies.^22^ The first method was used for the effects of sulfonylureas on volatile and amino acid contents, whereas the second method was used to investigate the recovery of sulfonylurea-treated fruits after precursor application.

In the first method, mature green ‘Valery’ fruits were cut into ∼1 cm^2^ sections, 2 mm thick, from transverse sections of fruits not treated with ethylene. Care was taken such that one edge of the square was from the edge of the pulp, thus representing an undisturbed or “live edge” of cells that should be able to maintain unhindered gas exchange. Pulp sections were prepared in vials as described above for apples. Treatment solution: 0.5 mM halosulfuron-methyl (methyl 3-chloro-5-(4,6-dimethoxypyrimidin-2-ylcarbamoylsulfamoyl)-1-methylpyrazole-4-carboxylate, made from Sandea, Gowan Co. 75% w/w active ingredient; 0.1% Tween 20). Ripening was induced by injection of 1 μL of ethylene into the vial’s 20 mL headspace. The following day, the vials were vented for 15 min before having the Mininert valve (Valco Instruments Co. Inc., Houston, TX) replaced on the vial but with a needle inserted to allow for gas diffusion. Daily for the following 2 days, 20 μL of a freshly made inhibitor or water solution was added onto the pulp. The needles were again kept in the valves to maintain gas diffusion. The next day, and thus 4 days since initially preparing the vials, the needles were removed, and the vials were sealed and incubated for at least 1 h at room temperature (22 °C) before headspace sampling.

While the results of inhibition using this methodology were consistent in ripening fruits, maintaining the small pulp sections at an appropriate humidity to allow for ripening while also preventing desiccation and microbial outbreak proved to be exceedingly difficult. Thus, a second methodology was employed. In this case, whole mature green banana fruits were gassed with 100 μL·L^–1^ ethylene for 24 h at 22 °C to trigger ripening. The following day, fruits were sanitized with a mild 5% bleach solution, rinsed with water, wetted with a 95% ethanol solution, and dried under a laminar flow sterile hood. Transverse 1 cm-thick banana disks including peel were excised and infiltrated with AHAS inhibitors under aseptic conditions. Rimsulfuron was applied at 0.025 and 0.0125 mM, imazamox was applied at 0.125 and 0.5 mM, and halosulfuron-methyl was applied at 0.0125 and 0.05 mM. To infiltrate, the discs were mounted in 60 mL glass Buchner funnels with either coarse or medium frit filters. The surface of the frit was covered with a filter paper disk (Whatman, No. 2), and a seal was made between the tissue disk and the inner wall of the funnel using a warm 1% agar solution, which, as it cooled, was built up to form an elevated surface around the disk. The upper surface of the peel tissue was sealed with a layer of agar to restrict the flow of the inhibitor to the pulp tissues only. Care was taken to prevent the agar from seeping under the disk and blocking the filter paper pores. The inhibitor solution (2 mL) was applied to the upper surface of the disk and vacuum (<5 mm Hg) was applied for 3 to 5 min until all the applied solution was pulled through the disk and the visible surface of the disk appeared dry. The disks were removed from the funnels and cleaned of residual agar before transfer to filter paper disks in sterile glass canning jars (480 mL). The jars were sealed with a metal lid in which a 7 mm diameter rubber septum was mounted. Disks were treated with 100 μL·L^–1^ ethylene, and jars were vented daily to minimize CO_2_ buildup and O_2_ depletion. Volatile analysis was performed 4 days after the initiation of ripening. This method, when fresh fruits were used, led to more reliable ripening with less maintenance than the first method and yielded the same volatile content shifts.

To study complementation, small sections of pulp were prepared as described above with 0.025 mM rimsulfuron or water and fed 80 μL of aqueous solutions containing 4 mM α-ketoisovalerate, 4 mM α-ketoisocaproate, 20 mM valine, or 20 mM leucine. The different α-ketoacid and amino acid concentrations were used to obtain comparable recoveries of the downstream esters.

### Volatile Analysis

Headspace volatiles from small tissue samples incubated in 22 mL vials were adsorbed for 30 s using a solid-phase microextraction (SPME) fiber (65 μm PDMS-DVB; Supelco Analytical, Bellefonte, PA). The SPME fiber was then directly desorbed for 1 min in the injection port of a gas chromatograph (GC; HP-6890, Hewlett-Packard, Wilmington, DE) coupled to a time-of-flight mass spectrometer (MS; Pegasus II, LECO, St. Joseph, MI). Whole fruits and banana slices were incubated for 20 min at room temperature (22 °C) in 1 L sealed Teflon jars before a 15 s or 3 min adsorption and 1- or 2 min desorption. Adsorption times were adjusted to ensure that instrument responses were in the linear range. All desorbed volatiles were cryofocused at the beginning of the column by immersing the said region of the column in liquid nitrogen. After the desorption period, the run was initiated, and the liquid nitrogen was removed.

The conditions of the system were as follows. Injection port: 200 °C, splitless, ultrapurified helium (99.999%) carrier gas, front inlet flow 1.5 mL·min^–1^ constant, 10 mL·min^–1^ purge flow, 11.5 mL·min^–1^ total flow. Oven: initial temperature 40 °C for 0 min, ramped by 43 °C·min^–1^ to 185 °C for 0 min. Column: HP-5MS, 30 m × 0.25 mm inner diameter, 0.25 μm film thickness (Agilent, Santa Clara, CA). Transfer line temperature, 225 °C. MS: Electron ionization (−70 eV), ion source temperature 200 °C, solvent delay 50 s, *m*/*z* range 29 to 400, detector voltage 1500 V, data collection rate 20 Hz.

When α-ketoisocaproate was supplied to apple fruits and separation of 2-methylbutyl acetate and 3-methylbutyl acetate was of interest, the following oven parameters were used: initial 40 °C for 0 min, 10 °C·min^–1^ until 100 °C, 20 °C·min^–1^ until 130 °C, and 60 °C·min^–1^ until 185 °C.

Compounds were identified by comparison of the retention time and mass spectrum against authenticated reference standards and spectra (National Institute of Standards and Technology Mass Spectral Search Program, Version 2.0, 2001). Volatiles were quantified by calibration with a standard of 59 authenticated, high-purity compounds (Table S2) (Sigma-Aldrich Co., St. Louis, MO, and Fluka Chemical, Seelze, Germany) including the target compounds. The standard was made by placing 0.5 μL of an equal-part mixture of the neat compounds onto a disc of filter paper before quickly placing the filter paper into a 4 L sealed flask fitted with a Mininert valve for SPME fiber access, as described previously,^23^ and allowed to vaporize for at least 6 h. SPME adsorption and desorption times of the standard matched those of the samples quantified (e.g., 30 s adsorption, 1 min desorption when quantifying samples in 22 mL vials). The quantification *m*/*z* of each compound can be seen in Table S3.

After volatile analysis, apple peel disks, collected peel of flowering quince, and ‘Valery’ pulp section samples were held at −80 °C for further amino acid analysis.

### Validation of SPME Methodology

A series of standard mixtures composed of 68 authenticated, high-purity alcohols, esters, and estragole were prepared (Table S2). One mixture contained all 68 compounds in equal-volume parts, the other mixtures were prepared such that 66 of the compounds were maintained at the same concentration, whereas two esters, 1-methylbutyl acetate and 1-methylbutyl butanoate, were selectively diluted to 50, 20, 10, 5, 2, and 1% of their original concentrations. Headspaces of these mixtures were prepared in 4 L flasks as described above. Adsorption times were 30 s or 3 min, and cryofocused desorption periods were kept at 2 min. GCMS parameters, compound identification, and compound quantification were as described above.

The logarithmically scaled relationship between the diluted ester concentrations and instrument response was linear regardless of the adsorption period (*R*^2^ > 0.996) (Figure S3). The slopes (average of 1.081) are very close to their theoretical value (1.000) and are within the expected margin of error when handling μL quantities of liquids. This error is also much less than the biological variation observed herein. Thus, in this case, diluted compounds among a relatively concentrated background can be reliably quantified without the influence of matrix effects.

We furthermore assessed the effect of adsorption time using the full-strength 68 compound mixture (Figure S3). Longer adsorption periods, in this case, greater than 150 s, led to an apparent matrix effect as the response of lower molecular mass compounds was displaced by heavier ones. However, if the adsorption period was strictly consistent, there was no impact on response linearity, indicating no meaningful impact for quantification at either 30 or 180 s, as has been previously demonstrated.^23^

### Amino Acid Analysis via UPLC-MS/MS

Frozen samples were ground to a powder in a liquid-nitrogen-chilled mortar and pestles. About 500 mg of tissue was vortexed for 10 s in 2 mL of room-temperature (22 °C) extraction solution (1:1:1 water: acetonitrile: ethanol, v/v/v) spiked with 2 nmoles of U–^13^C,^15^N labeled amino acids (MilliporeSigma, Bellevue, WA) as internal standards and heated for 15 min in a 65 °C water bath. Extracts were briefly chilled on ice before centrifugation at 4400*g* for 15 min at 4 °C. The supernatant was filtered by centrifugation (0.2 μm nylon centrifugal filter; Costar, Corning) at 21,000*g* for 5 min at room temperature. Ten μL of the filtrate was transferred to an autosampler vial and diluted 100-fold with 990 μL of 10.1 mM perfluorohexanoic acid (PFHA) spiked with 2 μmoles of internal standard. Thus, the final concentration of the internal standard was ∼2 μM.

An amino acid standard series was prepared from a premade mixture (MilliporeSigma, AAS18) that contained equal molar amounts of cystine and all 20 proteinogenic amino acids, except for tryptophan, asparagine, glutamine, and cysteine. An equal molar mixture of tryptophan, asparagine, glutamine, and cysteine was subsequently prepared. To avoid dilution errors or artifacts from differing buffers, these amino acid stocks were aliquoted and desiccated such that a five-part standard series ranging from 250 μM to 25 nM would be produced upon resuscitation with 10 μL of spiked extraction buffer and 990 μL of spiked PFHA solution.

Samples and amino acids were held overnight at −20 °C before analysis. Amino acids were analyzed with a Xevo TQ-S Micro UPLC (H-Class)-MS/MS (Waters, Milford, MA) instrument at the Michigan State University Mass Spectrometry and Metabolomics Core. Conditions were as follows. HPLC column: Acquirt UPLC HSS T3, 2.1 mm × 100 mm, 1.7 μm particle size (Waters), with a 0.2 μm precolumn filter (Waters). Mobile phase: (A) 10 mM PFHA in water and (B) acetonitrile. LC gradient: linear gradient, slope setting = 6, flow rate = 0.3 mL·min^–1^, step (1) 0 min, 100% A, 0% B, (2) 1 min, 100% A, 0% B, (3) 8 min, 35% A, 65% B, (4) 8.01 min, 10% A, 90% B, (5) 9 min, 10% A, 90% B, (6) 9.01 min, 100% A, 0% B, (7) 13 min, 100% A, 0% B. Column temp: 40 °C. Autosampler temp: 10 °C. Injection volume: 10 μL. Tune parameters: electrospray ionization, standard ESI probe, capillary voltage = +1.0 kV, source temp = 120 °C, desolvation temp = 350 °C, desolvation gas = 800 L·hr^–1^, cone gas = 40 L·hr^–1^. MS collection was split into three phases and was adjusted after checking the retention time of several samples; however, proline was missed for apple and flowering quince samples. Parent and daughter ions, cone and collision voltages, phases collected, and approximate retention times can be seen in Table S4. Quantification was performed by comparison of the ratios of peak areas of metabolites to labeled internal standards.

### Precursor Analysis via Derivatization and GCMS

To simultaneously quantify branched-chain α-ketoacids and amino acids, 100 mg of ground frozen tissue was extracted in 2 mL of 1:1 acetonitrile: water containing 13 μM U–^13^C, ^15^N-labeled amino acids (MilliporeSigma) as an internal standard for 15 min in a 65 °C water bath. Extracts were briefly chilled on ice before being centrifuged at 4400*g* for 10 min at 4 °C. The supernatant was transferred to a microcentrifuge tube and further centrifuged at 21,000*g* for 10 min prior to syringe filtration (0.45 μm, 13 mm diameter nylon filters with polypropylene syringes; Whatman, Cytiva). Precisely, 1.5 mL of the filtrate was alkalized to pH > 7.5 with 50 μL of 1 M NaOH, verified with 50 μL of 1% w/v 4-nitrophenol. Samples were desiccated overnight via rotovac (DNA100 Speed Vac, Savant, Hyannis, Mass.) at 22 °C. Samples were first derivatized via methoxyamination by addition of 100 μL of 40 mg·mL^–1^ methoxyamine hydrochloride in anhydrous pyridine and incubation for at least 12 h at 60 °C. They were next derivatized via *tert*-butyldimethylsilyation by addition of 100 μL of *N*-methyl-*N*-*tert*-butyldimethylsilyl trifluoroacetamide containing 1% *tert*-butyldimethylsilyl chloride and incubation for at least 12 h at 60 °C. Derivatized samples were centrifuged at 21,000*g* for 5 min before transferring the supernatant into autosampler vials. If the analysis was delayed, derivatized samples were held at −20 °C.

A one microliter portion of the derivatized sample was analyzed with GC (Agilent 6890, Agilent, Santa Clara, CA) coupled to time-of-flight MS (Pegasus II, LECO, St. Joseph, MI). The conditions of the system were as follows: injection port: 250 °C, 5:1 split, helium carrier gas, 7.5 mL·min^–1^ split flow, 9 mL·min^–1^ total flow. Oven: initial temperature at 80 °C for 0 min, ramped by 30 °C·min^–1^ to 130 °C for 0 min, then ramped at 15 °C·min^–1^ to 250 °C for 0 min, and then ramped at 40 °C·min^–1^ to 300 °C for 4 min. Column: VF-5 ms, 30 m × 0.25 mm i.d., 0.25 μm film thickness (Agilent, Santa Clara, CA). Transfer line temperature 300 °C. MS: Electron ionization (−70 eV), ion source temperature 230 °C, scanning 29–600 u, 20 spectra·s^–1^, 1800 acquisition voltage. Relatively high citramalate levels necessitated rerunning samples at a 20:1 split to avoid saturation of the detector.

Compounds were identified by retention time and spectra of derivatized authenticated standards (Table S5). Quantification was performed by comparison of the ratios of peak areas of metabolites to labeled internal standards. Among compounds that did not have a labeled internal analogue present, peak area ratios were compared against a labeled amino acid: α-ketoacids to valine and citramalate and α-isopropylmalate to isoleucine. Calibration standards consisted of equimolar mixtures of the analyzed compounds that mimicked 15–0.15 nmoles of the analyte in the final derivatized sample.

Great care was taken in the accurate quantification of α-keto-β-methylvalerate. Methoxyamination results in an imine whose cis–trans isomers may chromatographically separate. Furthermore, the chiral carbon of α-keto-β-methylvalerate racemizes under alkaline conditions. Under our analytical conditions, derivatized α-keto-β-methylvalerate manifests as a triplet. To counter this dilution of signal, the detector voltage was increased from routine conditions (1500 to 1800 V).

While results from the derivatization of banana tissue are not reported herein, it is worthwhile mentioning that tissues with a higher sugar content, such as banana fruit pulp, necessitate greater amounts of derivatizing reagent. We have found that 400 μL of each reagent per 100 mg of banana tissue is a good compromise.

### Statistical Analysis

Statistical analysis was performed using SciPy (v1.6.2), RStudio (v2023.03.0 + 386), or Microsoft Excel (v16.89). Against a specified value: one-tailed *t*-test. Against two treatments: paired *t*-test for banana, two-sample *t*-test for apple, and flowering quince. Against more than two treatments: Tukey’s test following significant ANOVA. Analyses of branched-chain amino acids were one-tailed; all other metabolites were two-tailed. The exogenous feedings of metabolites led to unequal variance; in these cases, data was transformed via log(*x* + 1) before statistical analysis. In all analyses, α = 0.05. The percent of metabolite content of treated tissues against control was calculated for each sulfonylurea-treated sample against the average of controls for apples and flowering quince (unpaired samples), and for bananas, it was calculated against the paired control sample.

## Results

### Experimental Philosophy and Methodology

To test our hypotheses, we treated apples, flowering quince, and banana fruit with inhibitors of acetohydroxyacid synthase (AHAS, also known as acetolactate synthase; Figures 1 and 2). AHAS is the common enzyme of branched-chain amino acid and α-ketoacid biosynthesis. It can be inhibited by compounds from a number of chemical families. Among them, sulfonylureas and imidazolinones have been determined to act by binding within and obstructing the substrate channel that leads to the active site of AHAS,^24^ resulting in a loss of catalytic activity. Given the lack of an alternative biosynthetic pathway, AHAS inhibition arrests *de novo* branched-chain α-ketoacid and amino acid biosynthesis, ultimately translating into severe inhibition of DNA synthesis, a halt of mitosis, and eventual plant death,^25^ hence, their widespread application as commercial herbicides.

Treatment with AHAS inhibitors can lead to variable changes (including no change) of *AHAS* gene expression in different plant species/tissues.^26−28^ However, any potential transcriptional or translational changes are inconsequential, as whatever AHAS present will be deactivated by the inhibitor. Complementation with knocked-down ester precursors will assess whether other elements of the biochemical pathway have been compromised by toxic side effects.

When applying these inhibitors, fruits at or near the onset of ripening were intentionally selected for use (phrased as “ripening”) for most experimental runs. In these cases, treatment began prior to accumulation of branched-chain amino acids. For apples, the peel is the primary site of aroma biogenesis and the location of the greatest accumulation of branch-chain amino acid (i.e., isoleucine).^14,29^ For bananas, the pulp is the primary source of branched-chain esters and the location of the greatest accumulation of branched-chain amino acids (i.e., valine and leucine)^16^. Using fruit at an early ripening stage catches the fruit before a substantial pool of branched-chain precursors have accumulated. To assess fruits that have already accumulated a supply of precursors, an inhibitor was applied to ‘Red Delicious’ apple fruits of advanced ripeness (phrased as “ripe”).

Methodology trials with apple and banana fruits using sulfonylureas and imidazolinones led to marked decreases of the headspace content of *anteiso*- and *iso*-branched-chain esters (Figure S2A). The replicated effects from the application of these distinct chemical families, as well as subsequent results from amino acid content analyses described herein, assured us that these well-studied compounds were acting as inhibitors of AHAS and that the observed results were not due to fruit- or compound-specific idiosyncrasy. Further experimentation was performed with sulfonylureas.

### Apple

**Figure 3:**
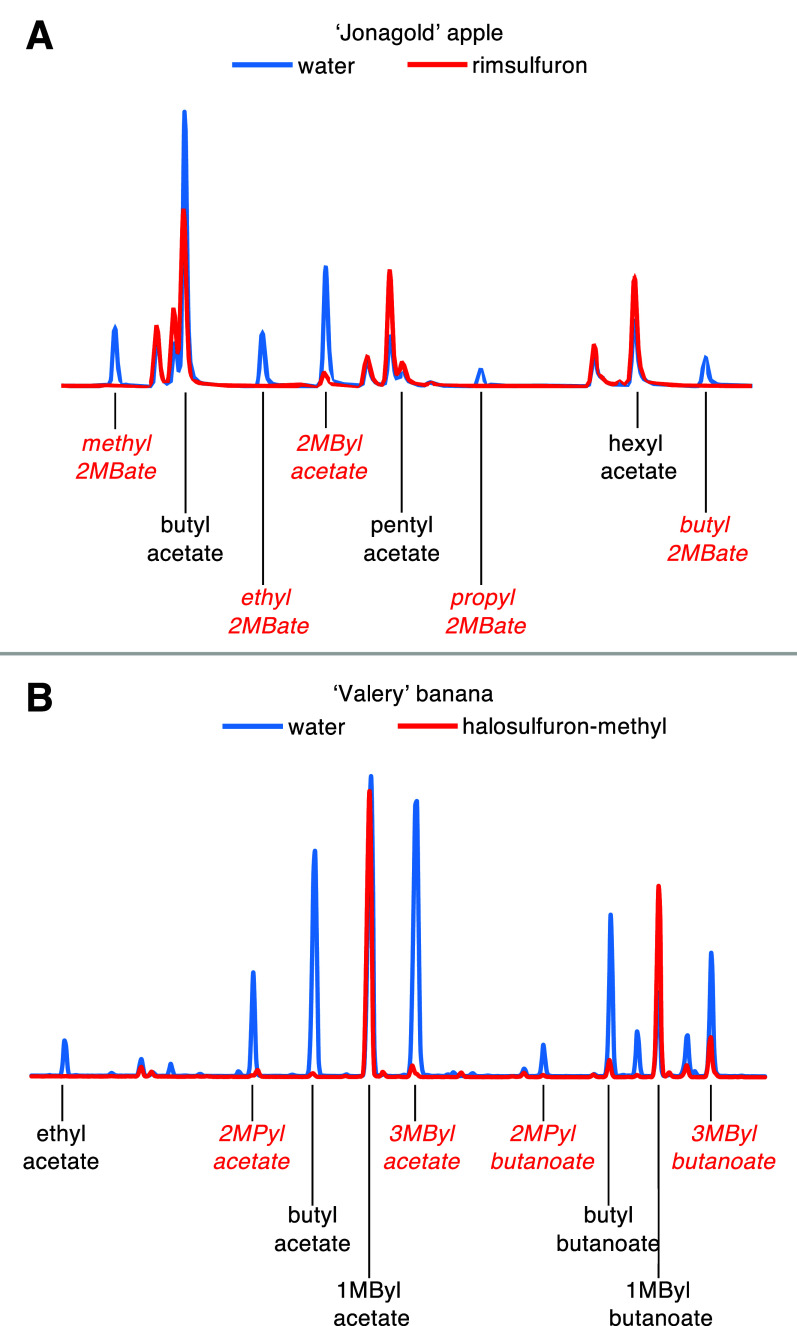
Representative total ion chromatogram sections from incubation chamber headspace of apple and banana fruit tissues treated with water or an acetohydroxyacid synthase inhibitor. *Anteiso*- and *iso*-branched-chain esters highlighted in red. (A) ‘Jonagold’ apple fruit peels treated with water or rimsulfuron and fed methanol. (B) ‘Valery’ banana fruit pulp sections treated with water or halosulfuron-methyl. 1MByl = 1-methylbutyl, 2MBate = 2-methylbutanoate, 2MByl = 2-methylbutyl, 2MPyl = 2-methylpropyl, 3MByl = 3-methylbutyl.

The application of rimsulfuron, a sulfonylurea AHAS inhibitor, led to the significant reduction of the total content of *anteiso*-branched-chain esters in the headspaces of ripening ‘Gala’, ‘Empire’, and ‘Jonagold’ apple fruits by 91.2% on average, with every 2-methylbutyl and 2-methylbutanoate ester analyzed having decreased (Figure 3A; Table 1; Figures S4 and S5; Table S6). Furthermore, 2-methylpropyl acetate, which is present in quantities of less than 2 nmol·L^–1^ in the headspaces of all three cultivars, was also reduced following treatment. While rimsulfuron treatment had no discernible effect on butyl acetate, the concentration of pentyl acetate, on average, was 1.63-fold more abundant in all three of the cultivars when treated. No consistent pattern of change was observed in the other straight-chain esters.

**Table 1:** Percent of Acetate Ester and Total *Anteiso*- and *Iso*-Branched-Chain Ester Emissions of Sulfonylurea-Treated Fruit Tissues Compared to Control^a^

	apple peel			banana pulp	flowering quince peel
acetate ester	‘Gala’	‘Empire’	‘Jonagold’	‘Valery’	‘Dr. Banks Pink’
ethyl acetate	57.7 ± 9.3	61.4 ± 7.9	–	3.9 ± 2.4	...
propyl acetate	–	53.8 ± 8.5	–	...	2265.0 ± 869.0
butyl acetate	–	–	–	3.9 ± 2.7	–
pentyl acetate	155.3 ± 17.5	161.9 ± 16.0	172.3 ± 20.2		
hexyl acetate	–	–	162.7 ± 19.2	...	
1-methylbutyl acetate				–	
**2-methylbutyl acetate (ile)**	**27.0 ± 6.3**	**1.9 ± 0.6**	**16.1 ± 1.4**		**5.2 ± 3.9**
**2-methylpropyl acetate (val)**	36.4 ± 5.0	27.3 ± 5.4	67.6 ± 4.1	**2.0 ± 0.7**	**1.1 ± 1.4**
**3-methylbutyl acetate (leu)**				**10.4 ± 5.4**	
**sum of ***anteiso***- and ***iso***-branched-chain esters**	**19.0 ± 4.5**	**0.8 ± 0.2**	**6.5 ± 0.5**	**9.7 ± 3.6**	**3.0 ± 2.4**

a Percent presented if the content of treated tissues was significantly different (two-tailed two-sample equal variance *t*-test for apple and flowering quince, paired *t*-test for banana; α = 0.05). Presented as means ± SE of sulfonylurea-treated samples against the average of controls for apple and flowering quince (unpaired samples) and for banana as the ratio of paired samples. Major *anteiso*- and *iso*-branched-chain esters highlighted in bold with related amino acids listed parenthetically. Note that *iso*-branched-chain esters are normally present in near trace amounts in apple fruit aroma. Dash indicates no significant difference between treatments, ellipsis indicates present volatiles that were not quantified, and blank indicates volatiles that are not present. For quantified levels of these and other volatiles, as well as *p*-values, see the Supporting Information.

Isoleucine was significantly reduced, on average, by 92.1% in the peels of ripening fruit for the three cultivars after rimsulfuron treatment (Tables 2 and S7–S9). Interestingly, among the other amino acids dependent upon AHAS for synthesis, valine was unaffected following treatment, whereas the content of leucine increased, on average, 3.4-fold in the three ripening cultivars. Notably, however, no other amino acid was reduced following treatment. While several were found to be somewhat elevated, no pattern of change among nonbranched-chain amino acids was consistent across the ripening cultivars. Only the ‘Gala’ fruit had a significant increase of total free amino acid content after treatment.

**Table 2:** Percent of Free Amino Acid Content of Sulfonylurea-Treated Fruit Tissues Compared to Control^a^

	apple peel			banana pulp	flowering quince peel
amino acid	‘Gala’	‘Empire’	‘Jonagold’	‘Valery’	‘Dr. Banks Pink’
alanine	273.4 ± 27.4	–	–	407.4 ± 66.8	–
arginine	–	–	–	–	–
asparagine	–	–	–	–	–
aspartate	231.8 ± 18.0	–	–	332.7 ± 30.1	–
cysteine	–	261.5 ± 32.5	410.6 ± 49.6	264.2 ± 46.6	–
glutamate	146.3 ± 5.6	–	–	217.4 ± 23.9	–
glutamine	250.7 ± 33.5	–	–	–	–
glycine	–	–	–	173.1 ± 39.9	–
histidine	–	–	–	–	166.5 ± 20.1
**isoleucine**	**16.4 ± 6.3**	**3.3 ± 0.7**	**4.1 ± 1.3**	–	**51.3 ± 9.1**
**leucine**	214.4 ± 42.2	422.8 ± 100.1	393.2 ± 74.4	**65.8 ± 6.1**	81.9 ± 5.4
lysine	–	–	–	–	–
methionine	–	212.7 ± 26.8	218.3 ± 31.0	–	245.3 ± 38.5
phenylalanine	–	–	–	–	–
proline	...	...	...	188.4 ± 18.7	...
serine	300.1 ± 27.7	–	191.9 ± 32.1	186.2 ± 14.5	302.1 ± 32.0
threonine	182.3 ± 14.2	–	–	–	–
tryptophan	–	–	–	–	–
tyrosine	–	–	–	142.3 ± 25.1	–
**valine**	–	–	–	**14.3 ± 0.8**	**28.1 ± 3.8**
total	191.5 ± 20.4	–	–	150.6 ± 13.7	–

a Percent presented if the content of treated tissues was significantly different (two-sample equal variance *t*-test for apple and flowering quince, paired *t*-test for banana; one-tailed test for valine, leucine, and isoleucine; two-tailed test for all other amino acids; α = 0.05). Presented as means ± SE of sulfonylurea-treated samples against the average of controls for apple and flowering quince (unpaired samples) and for banana as the ratio of paired samples. Branched-chain amino acids with related volatiles present in headspace highlighted in bold. Dash indicates no significant difference between treatments, and ellipsis indicates amino acids that were not quantified. For quantified amino acid levels and *p*-values, see the Supporting Information.

**Figure 4:**
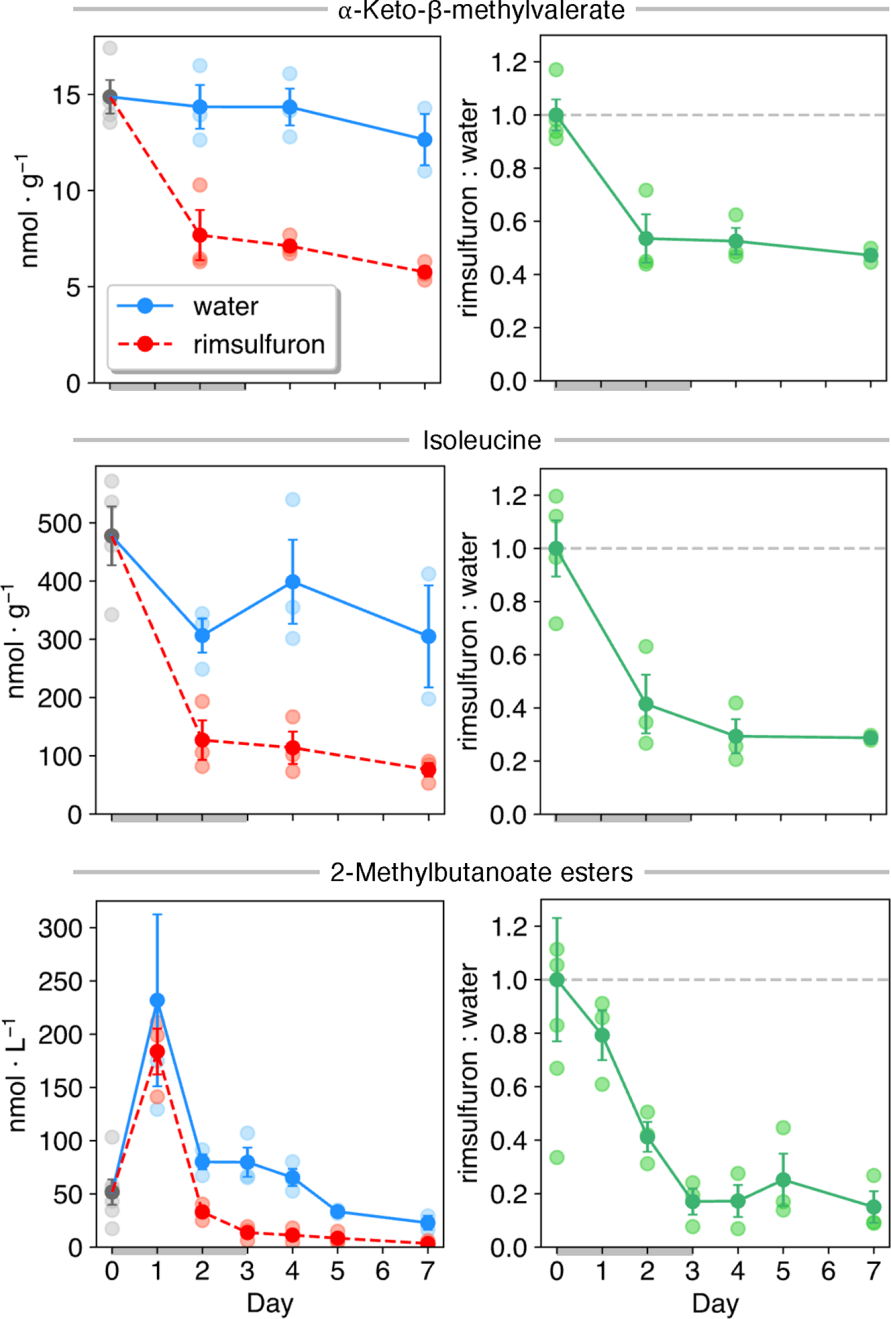
Fruit headspace and peel tissue content of *anteiso*-branched-chain esters and their precursors, respectively, of ripe ‘Red Delicious’ apple fruits treated with rimsulfuron over time. 2-Methylbutanote ester content is the sum of ethyl 2-methylbutanoate, butyl 2-methylbutanoate, and hexyl 2-methylbutanoate. The gray bar indicates the treatment period (d 1–3). Ratios calculated by comparing rimsulfuron-treated samples against the average of controls on the said day or, for initial samples, against the average of all samples. All means were found to be significantly different except for headspace content and ratio of 2-methylbutanoate esters on d 1 (two-tailed for esters, one-tailed for acids, two-sample equal variance *t*-test for metabolite content; two-tailed one-sample *t*-test for ratios; α = 0.05). Data presented as means ± SE of ≥ three biological replicates.

To help discern the metabolic flux of these processes, ripe fruits that had already accumulated a substantial pool of available precursors were treated with rimsulfuron. At the start of treatment, the peel tissues of ripe ‘Red Delicious’ apple fruits used had nearly 500 nmol·g^–1^, collectively, of isoleucine and it is α-ketoacid, α-keto-β-methylvalerate (Figure 4). Within 2 days of inhibitor application, the content of these precursors had dropped considerably, eventually plateauing to 29.1% and 51.1% of control levels, respectively. The headspace content of 2-methylbutanoate esters likewise rapidly decreased to 15.0% of control fruits within 3 days of treatment. Thus, even ripe fruits that have amassed a substantial pool of precursors appear to rely heavily upon *de novo* replenishment of this evidently rapidly turned-over supply.

**Figure 5:**
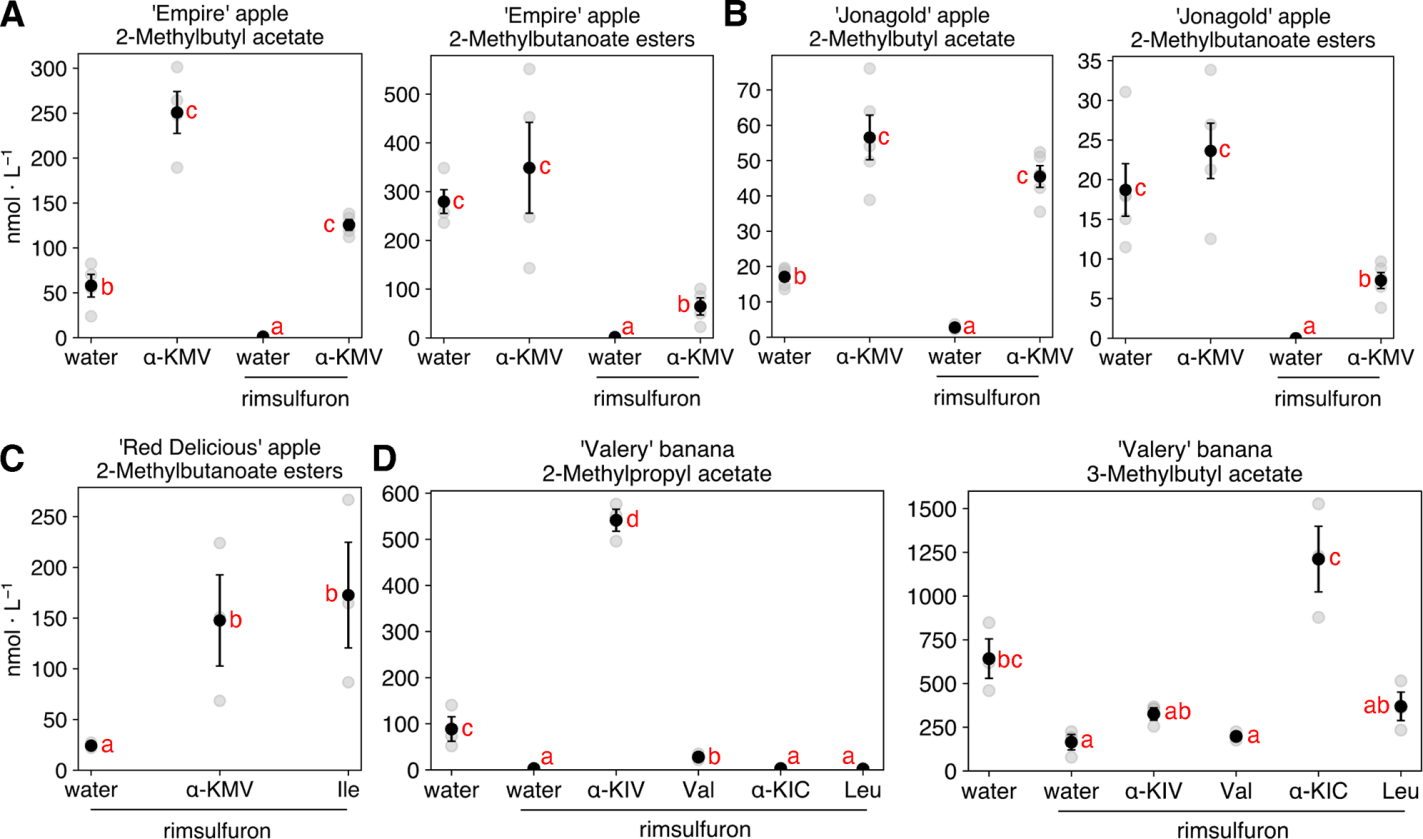
Effects of rimsulfuron on *anteiso*- and *iso*-branched-chain ester headspace content in fruits and complementation with precursors. (A) Ripening ‘Empire’ apple fruit. (B) Ripening ‘Jonagold’ apple fruit. (C) Ripe ‘Red Delicious’ apple fruit. Feedings performed on day seven of the time course and measured the next day. (D) Ripening ‘Valery’ banana fruit. 2-Methylbutanote ester content is the sum of ethyl 2-methylbutanoate, butyl 2-methylbutanoate, and hexyl 2-methylbutanoate. α-KMV = α-keto-β-methylvalerate, α-KIV = α-ketoisovalerate, and α-KIC = α-ketoisocaproate. Significantly different headspace concentrations are denoted by different letters adjacent to means (data transformed (log(*x* + 1)) for statistical analysis due to the unequal variance of fed samples; Tukey’s test, α = 0.05). Data presented as means ± SE of ≥ three biological replicates.

To determine if inhibitor-treated fruit were still capable of aroma production, branched-chain precursors were fed to the peel tissues of rimsulfuron and water-treated ripening ‘Empire’ and ‘Jonagold’ and ripe ‘Red Delicious’ apple fruits. Feedings of isoleucine or α-keto-β-methylvalerate to rimsulfuron-treated ripe ‘Red Delicious’ fruit peels resulted in recovery of *anteiso*-branched-chain esters (Figure 5C). As both precursors were found to provide effective recovery of branched-chain esters, further feedings in apples were carried out with only the branched-chain α-ketoacids. In ripening ‘Jonagold’ and ‘Empire’ fruits, exogenous feeding of α-keto-β-methylvalerate led to at least a partial rescue of all 2-methylbutyl and 2-methylbutanoate esters of the rimsulfuron-treated fruit (Figures 5A,B and S4 and S5). Together, these results indicate ester synthesis capability was sustained in inhibitor-treated fruit and that reduced branched-chain ester levels were a result of precursor scarcity, not inhibitor toxicity.

‘Jonagold’ and ‘Empire’ peel tissues were also capable of converting exogenous α-ketoisovalerate and α-ketoisocaproate, the α-ketoacids of valine and leucine, respectively, into an abundance of their respective *iso*-branched-chain aldehydes, alcohols, alkyl ester moieties (those derived from alcohols), and alkanoate ester moieties (those derived from acyl-CoAs). None of these compounds are normally produced in appreciable quantities by apple fruits but are abundant in bananas (Figures S4–S6; Table S10). Furthermore, the activity of isopropylmalate synthase was evident by the observed emanation of 3-methylbutanal, 3-methylbutanol, and several 3-methylbutyl esters from fruits fed with α-ketoisovalerate (Figure 1).

α-Keto-β-methylvalerate and α-ketoisovalerate were metabolized by both apple cultivars to similar molar concentrations of alkyl and alkanoate ester moieties. This is illustrated by the headspace concentrations of the respective ethyl and acetate esters of these α-ketoacids (Figures S4–S6). Interestingly, α-ketoisocaproate was metabolized into substantially fewer alkanoate ester moieties than alkyl ones, indicating nuance within the intermediary enzymatic steps between the α-ketoacids and their respective esters.

To aid in discerning modified carbon flow following rimsulfuron treatment, labeled (1,2-^13^C_2_) acetate was fed to apple fruits in conjunction with methanol (which allows for methyl ester production and the analysis of only newly synthesized esters).^17^ The fed acetate is readily metabolized into acetyl-CoA, a substrate of citramalate synthase for the production of branched-chain and straight-chain ester precursors (Figure 1).^17^

Incorporation of labeled acetate into ‘Gala’ was poor, likely resulting from acetate already being saturated in the treated tissues, as has been hypothesized to occur in other cultivars.^30^ While sufficient incorporation was observed in ‘Empire’ and ‘Jonagold’ tissues, methyl 2-methylbutanoate content was reduced to such a considerable extent by inhibitor treatment (99.4% and 100%, respectively) that the resulting instrument responses were too low for reliable analysis of isotope enrichment shifts. Among straight-chain esters, the M+1 and M+2 methyl pentanoate isotopologues were collectively 4.28-fold more enriched in both cultivars, on average, after treatment. In ‘Jonagold’ fruits, the M+1 isotopologue of methyl hexanoate also had slightly more enrichment than controls (Figure S7).

### Banana

The application of halosulfuron-methyl, another sulfonylurea AHAS inhibitor, caused a 90.3% reduction in the total content of *iso*-branched-chain esters in the headspace of ‘Valery’ banana fruit, with every *iso*-branched-chain ester and alcohol analyzed having decreased (Figure 3B; Tables 1 and S11). Surprisingly, ethyl acetate, butyl acetate, and butyl butanoate were also less in treated tissues (collectively reduced by 95.0%). None of the *sec*-branched esters or their related volatiles (2-pentanone, 2-pentanol, 1-methylbutyl acetate, and 1-methylbutyl butanoate) were affected by inhibitor treatment.

Valine and leucine were significantly less abundant (by 57.6%) in halosulfuron-methyl-treated fruits, whereas isoleucine levels were unchanged (Tables 2 and S12). While no other amino acid was reduced in treated fruits, several were found to be higher after inhibitor treatment with the total content of free amino acids having increased.

Similar to our results in apple fruits, exogenous application of precursors to tissues treated with the inhibitor led to a recovery of the *iso*-branched-chain esters (Figure 5D); however, the branched-chain α-ketoacids were considerably more effective than the amino acids. Banana fruits were also capable of metabolizing α-keto-β-methylvalerate into a variety of *anteiso*-branched-chain volatiles, as has been previously observed via the application of isoleucine (Table S13).^31^ While supplementation with the depleted precursors failed to recover ethyl acetate, butyl acetate, or butyl butanoate in this reported trial, we would be remiss to fail to mention that during method testing, we did occasionally observe a marked recovery of ethyl acetate and butyl acetate after α-ketoisocaproate application; further investigation is needed to better understand these observations.

### Flowering Quince

To demonstrate the reach of our hypothesis that *de novo* precursor synthesis is a major contributor to branched-chain ester biosynthesis in ripening fruits, we applied rimsulfuron to the highly aromatic fruit of an essentially uninvestigated ornamental quince hybrid (*Chaenomeles* ×*superba*, cv. Dr. Banks Pink; Figure S8).

The aroma profile of the small, dense fruit is dominated by the terpene linalool and the phenylpropene estragole, but low levels of the straight-chain esters propyl acetate, butyl acetate, and ethyl butanoate as well as the branched-chain esters 2-methylpropyl acetate and 2-methylbutyl acetate are present. As this species is a member of Maleae (the tribe of Rosaceae that includes apples and pears), it was assumed that the peel is the site of aroma biogenesis.^29^ Thus, rimsulfuron was applied to the peels of aroma-active fruits, and the effect on aroma production and amino acid content was analyzed.

Rimsulfuron-treated fruits had 94.6% less 2-methylpropyl acetate and 2-methylbutyl acetate but 22.7-fold more propyl acetate than untreated fruits. No difference was observed for butyl acetate, ethyl butanoate, estragole, or linalool (Tables 1 and S14).

Valine and isoleucine were collectively reduced by 64.2% in rimsulfuron-treated peel tissue (Tables 2 and S15). Leucine content was also reduced by 18.1% despite there being no leucine-related volatiles detected in the headspace of the fruit. No other amino acid was diminished by inhibitor treatment. The total content of free amino acids was not significantly altered by treatment.

## Discussion

The categorical suppression of all isoleucine-, valine-, and leucine-related volatiles by upward of 90% in the fruit tissues treated with an AHAS inhibitor robustly supports the hypothesis that these ripening fruit tissues rely heavily upon newly synthesized precursors to produce these important sensory compounds. Seemingly, only a very modest contribution is made from preexisting sources in the tested tissues, although there may be situations/fruits where they contribute more substantially than were observed herein. However, given that we likely did not achieve complete inhibition of AHAS (e.g., inhibition of plant AHAS by sulfonylureas plateaus at ∼85%^32,33^ and efficacy/penetration with the fruit tissues used is unclear), it is conceivable that virtually all *anteiso*- and *iso*-branched-chain ester synthesis is reliant upon *de novo* synthesized precursors in fruits.

As none of the fruits produce branched-chain esters that correspond to all three branched-chain amino acids, the parallel increases of the specific amino acids and their related volatiles in apple and banana fruits^14,16^ are seemingly due to specific portions of the branched-chain biosynthetic pathways undergoing programmed enhancements during ripening.

One of these enhancements seems to be the genetic expression of AHAS itself. Transcriptomic data sets indicate the expression of its catalytic subunit to be constitutively expressed throughout apple and banana fruit development with expression increasing 1.4-fold and 2.7-fold in ripening apple and banana fruits, respectively.^34,35^ However, since AHAS leads to the synthesis of all three branched-chain amino acids, other specific enhancements of the *anteiso*- or *iso*-branched pathway segments are necessary. In apples, this enhancement manifests as a 104.7-fold increase of citramalate synthase expression in ripening fruit peel during ripening,^34^ leading to the increased and nonfeedback regulated production of the AHAS substrate, α-ketobutyrate, for the synthesis of *anteiso*-branched-chain metabolites (Figure 1).

As shown herein, the formation of branched-chain ester moieties in apple fruits relies on precursors derived upstream of AHAS. This finding is entirely consistent with the characterization of apple cultivars lacking an enzymatically active allele of citramalate synthase; these cultivars do not accumulate substantial quantities of isoleucine or *anteiso*-branched-chain esters during ripening.^17^ Taken together, citramalate synthase appears to be critical for supplying the precursors of *anteiso*-branched-chain ester biosynthesis in apple fruits.

As inhibition of AHAS in apple fruits effectively eliminates the influx of carbon from α-ketobutyrate to branched-chain metabolism, flux should then be shunted toward further straight-chain elongation via citramalate synthase (Figure 1).^17^ The increased headspace concentrations and stable-isotope enrichment of pentyl acetate and methyl pentanoate, respectively, after rimsulfuron application is striking; however, it is unclear why there were not concomitant changes to other straight-chain esters as well. Further experimentation that targets the citramalate synthase pathway as well as “canonical” straight-chain ester biosynthesis pathways involving fatty acids will aid in better understanding the contributions of these sources to straight-chain ester biosynthesis in apple fruits.

Our results also demonstrate that apple and banana fruits, despite not normally producing appreciable amounts of all three classes of *anteiso*- and *iso*-branched-chain esters, have no general hindrance to synthesizing said esters when a supply of the corresponding α-ketoacids or amino acid is present. Thus, how precursor biosynthesis is engaged, such as *anteiso*-branched chain metabolism in apples or *iso*-branched chain metabolism in bananas, and what precursors accumulate in ripening fruits, plays an important role in determining a fruit’s resulting aroma profile.

In addition to the above inferences, the use of these inhibitors on banana fruit allows for the suggestion of the presence of a previously unknown means of butyl and butanoate ester biosynthesis. This appears to be unique to banana fruits since similar results were not observed in apple or flowering quince. Apart from some specialized instances of straight-chain 1-C elongation from α-ketobutyrate that have been documented in apple (via citramalate synthase^17^) and in *Solanaceae*,^36^ it has largely been assumed that butyl and butanoate esters are derived from β-oxidation of fatty acids: a catabolic process. As treatment of tissues with AHAS inhibitors does not lead to inhibition of fatty acid synthesis,^37^ our results suggest that the source of butyl and butanoate esters in banana fruit may originally be from branched-chain amino acid metabolism.

Some of the first scientific work that attempted to establish a relationship between leucine and 3-methylbutyl esters found that bananas fed with U–^14^C-leucine produced a significantly enriched volatile fraction containing butyl butanoate and 1-methylbutyl butanoate.^3^ In light of these results, we propose that α-ketoisocaproate may be the metabolite that banana fruits use to bridge branched-chain amino acid and butanoate metabolisms; however, additional work is needed to elucidate how this is facilitated.

The sulfonylurea-induced inhibition of ethyl acetate synthesis in banana fruit is surprising. However, another past study that likewise fed U–^14^C-leucine to ripening banana fruit found isotopic enrichment of the acetate moiety of 3-methylbutyl acetate,^1^ leading to the suggestion that even the precursors of ethyl acetate may be derived from branched-chain amino acid metabolism in banana fruit.

The lack of suppression of 2-pentanol, 2-pentanone, and 1-methylbutyl esters by the inhibitors strongly suggests that these *sec*-branched-chain compounds of banana fruit are derived from a source that is not within the sphere of AHAS’s influence. It is striking that banana fruit should produce multiple forms of branched-chain esters (*iso*- and *sec*-branched), which are of seemingly wholly independent origins.

In flowering quince, our results indicate that these fruits, like apple and banana fruit, rely largely upon *de novo* precursor synthesis to produce branched-chain volatiles. While our results cannot indicate whether the citramalate synthase pathway is present in flowering quince, the sole increase of propyl acetate after sulfonylurea application is, perhaps, telling. As propyl acetate is a potential product of α-ketobutyrate (Figure 1) and longer straight-chain products are not enhanced as they are in apple fruit, 1-C elongation of straight-chain α-ketoacids likely does not occur in flowering quince.

Ultimately, as branched-chain α-ketoacids are direct products of *de novo* synthesis, and if branched-chain esters are directly derived from branched-chain α-ketoacids,^14,15^ then interconversion of the amino acids and α-ketoacids may be unnecessary for ester biosynthesis. Thus, the substantial accumulation of branched-chain amino acids in these ripening fruits may be a byproduct of the enhancement of the biosynthetic branched-chain pathways, routed through AHAS, leading to branched-chain volatiles. On the other hand, in the later stages of ripening, it may also be that the accumulated branched-chain amino acids act as a storage reservoir that provides a buffering pool of available precursors. Regardless, as *de novo* synthesis of at least the branched-chain α-ketoacids is necessary for branched-chain volatile synthesis, we choose to refer to these volatiles as being “related to” branched-chain amino acids, rather than being “derived from” them.

Collectively, the use of AHAS inhibitors allows for rejection of the hypothesis that branched-chain esters are derived from preexisting branched-chain amino acids and α-ketoacids and instead supports the hypothesis that these esters are the product of *de novo* precursor biosynthesis. While inhibitors of general physiological processes, such as ethylene perception, protein synthesis, or respiration (via application of 1-methylcyclopropene, cycloheximide, potassium cyanide, arsenate, or 2,4-dinitrophenol), have been applied to ripening apple and/or banana fruit to study aroma production,^38−42^ we could find no other published work utilizing herbicidal enzymatic inhibitors to discern volatile biosynthetic pathways, despite their use in the investigation of many important aspects of plant metabolism and physiology.^43^ Future use of these and other enzymatic inhibitors on fruits should result in further advances in precursor production and aroma biochemistry in general.

Lastly, it is worthwhile pursuing the logical extension to our results. As we have demonstrated, ripening apple and banana fruits produce *anteiso*- and *iso*-branched-chain esters from newly synthesized precursors. However, the pathways that produce the said precursors are normally regulated by strict allosteric feedback mechanisms. The ability of these fruits to increase flux through these pathways during ripening seems paradoxical. While citramalate synthase explains how apple fruit circumvents such regulation, it is still unknown what regulatory changes occur in ripening banana fruits.

## Supporting Information Available

Description of apple fruit maturity indices, method parameters, headspace volatile concentrations and statistical results, and amino acid concentrations and statistical results (XLSX)
Structures of compounds discussed, results from method testing, headspace volatile concentrations and statistical results, results from feedings of apple peels with 1,2-^13^C_2_ acetate, and photos of flowering quince (PDF)

## Abbreviation

AHAS: acetohydroxyacid synthase, also known as acetolactate synthase.
