# Supplementary material for: Fruits Produce Branched-Chain Esters Primarily from Newly Synthesized Precursors
Source: J Agric Food Chem. 2025 Feb 7;73(7):4196–207. doi: 10.1021/acs.jafc.4c10677 (PMC11843720; doi:10.1021/acs.jafc.4c10677)
Supplement: Supplementary file 2 — jf4c10677_si_002.pdf [file jf4c10677_si_002.pdf]

Supplementary Figures

Fruit produce branched-chain esters primarily from newly synthesized precursors.

Philip Engelgau[a], Sumithra K. Wendakoon[b], Nobuko Sugimoto[a], and Randolph M. Beaudry[a]*

[a] Michigan State University, Department of Horticulture, East Lansing, MI, 48823, United States

[b] Ryukoku University, Department of Agricultural Sciences, Otsu, 520-2194, Japan

*Corresponding author email: beaudry@msu.edu, telephone: +1(517)353-0303

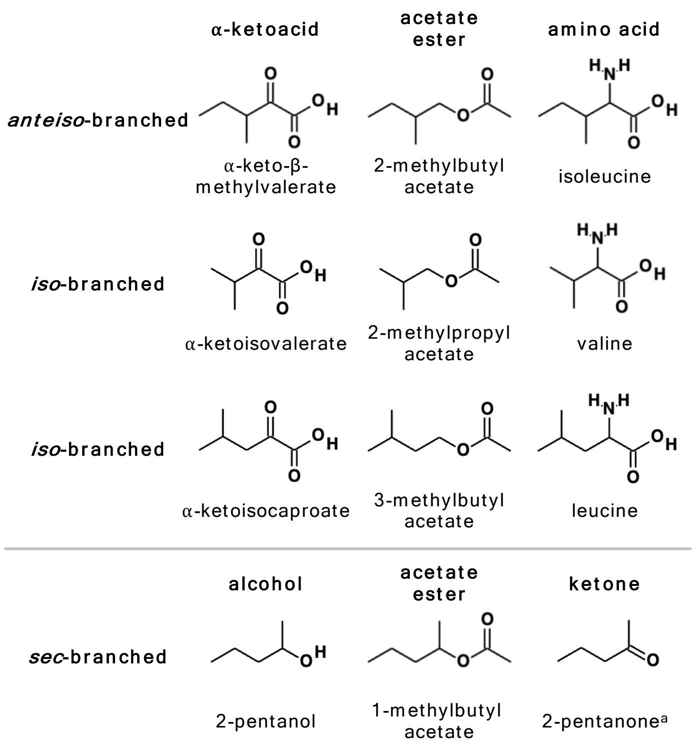

Supplementary Figure S1. Structures of some of the compounds discussed, including branching pattern designations. [a]2-Pentanone is not technically *sec*-branched but it is a product of 2-pentanol oxidation.

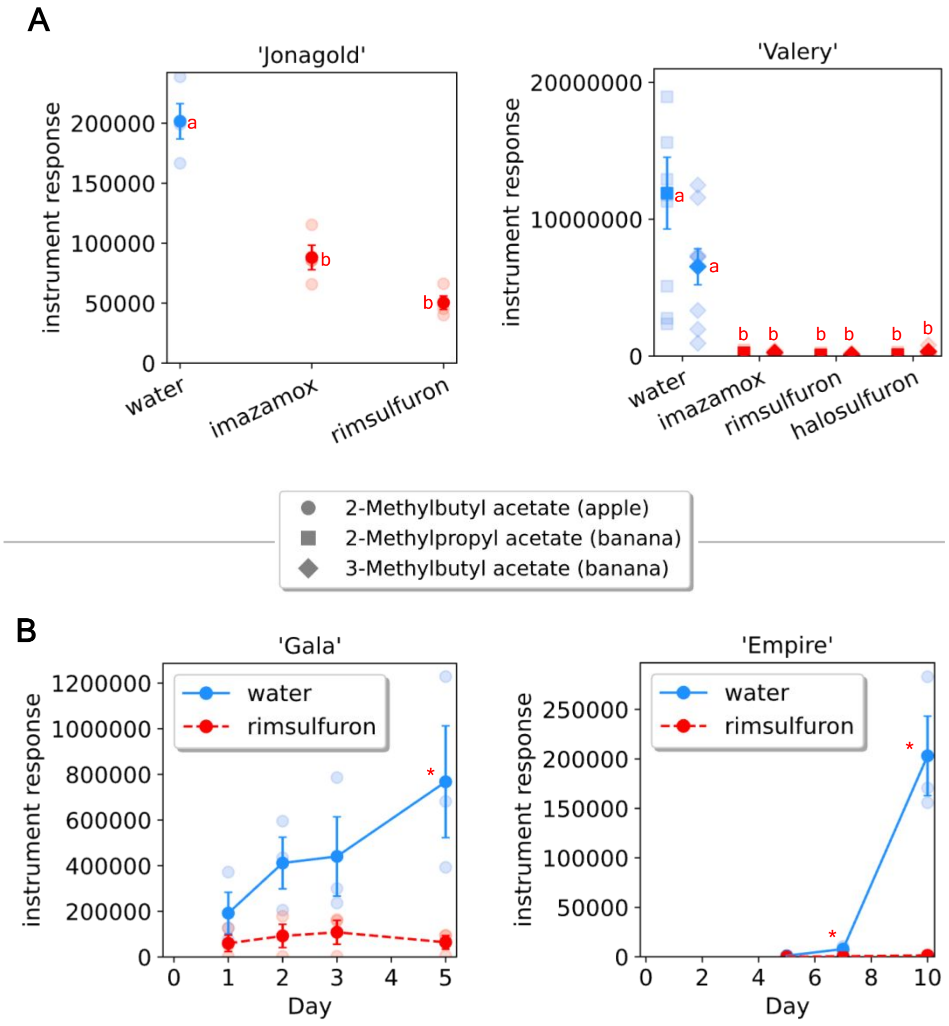

Supplementary Figure S2. Method testing with acetohydroxyacid synthase inhibitors and ripening fruits. A) Effects of various acetohydroxyacid synthase inhibitors on *anteiso*- and *iso*-branched-chain ester headspace content of apple and banana fruit. Imazamox is an imidazolinone. Rimsulfuron and halosulfuron (halosulfuron-methyl) are sulfonylureas. Significantly different instrument responses are denoted by different letters adjacent to means (data untransformed for apple, transformed (log(x+1)) for banana due to unequal variance of samples; Tukey's test, $\alpha = 0.05$) B) 2-Methylbutyl acetate headspace content of ripening apple fruit over time. Fruit were treated daily. Significantly different instrument responses are denoted by * adjacent to means (two-tailed two-sample equal variance t-test; $\alpha = 0.05$). All data presented as means ± SE of $\geq$ three biological replicates.

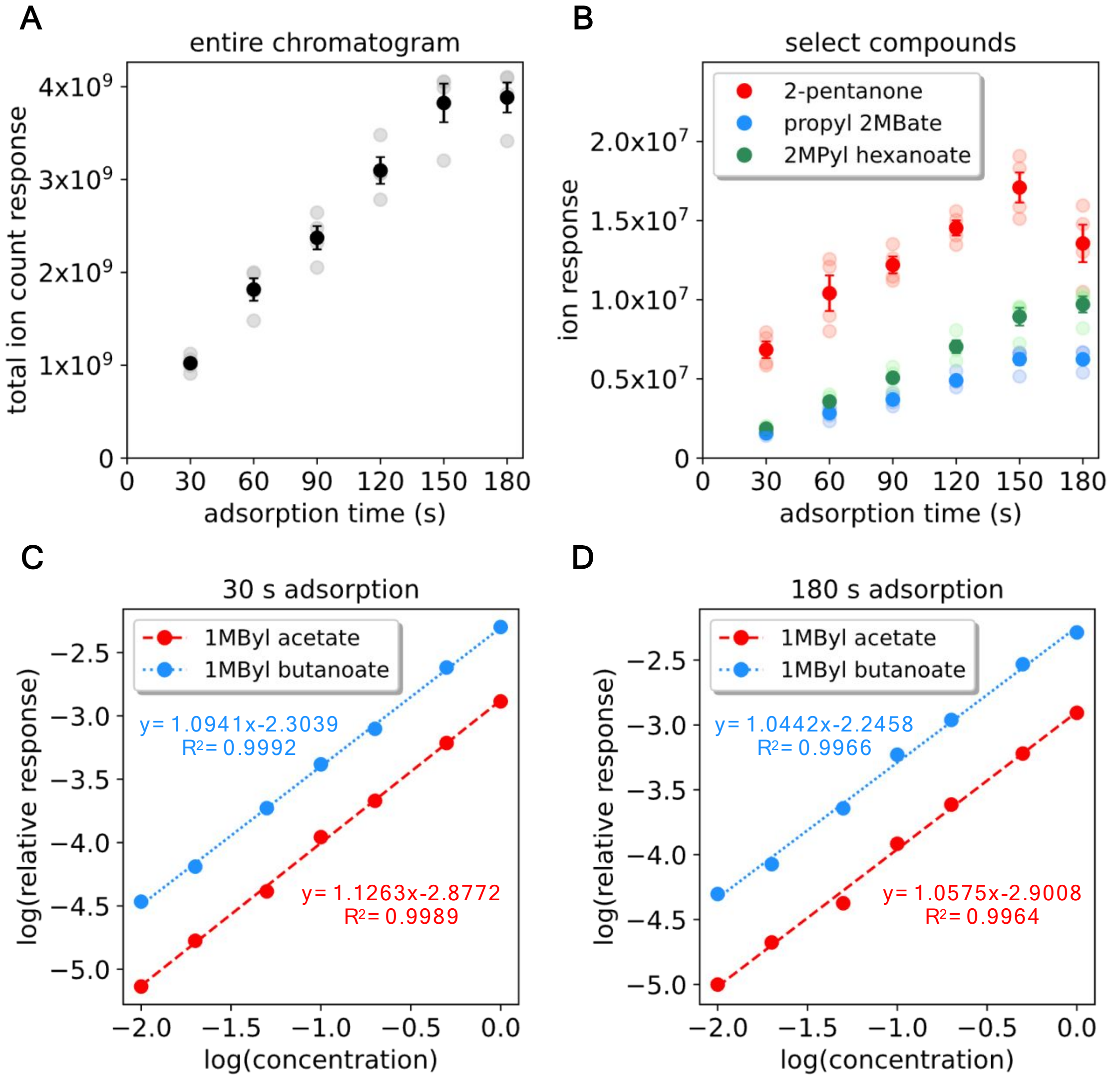

Supplementary Figure S3. Effects of SPME adsorption time and volatile concentration on response. A) Total ion count response of entire chromatogram vs adsorption time of 68 compound mixture. B) Responses of select compounds vs adsorption time of 68 compound mixture. C-D) Relative response (individual compound response:total ion count response of entire chromatogram) vs concentration of 2 compounds selectively diluted in a 68 compound mixture for two adsorption times. Fitted curves displayed. 1MByl = 1-methylbutyl, 2MBate = 2-methylbutanoate, 2MPyl = 2-methylpropyl. *m/z* used: 1MByl acetate = 87, 1MByl butanoate = 71, 2MPyl hexanoate = 99, 2-pentanone = 43, propyl 2MBate = 85. Data presented as means ± SE of four technical replicates.

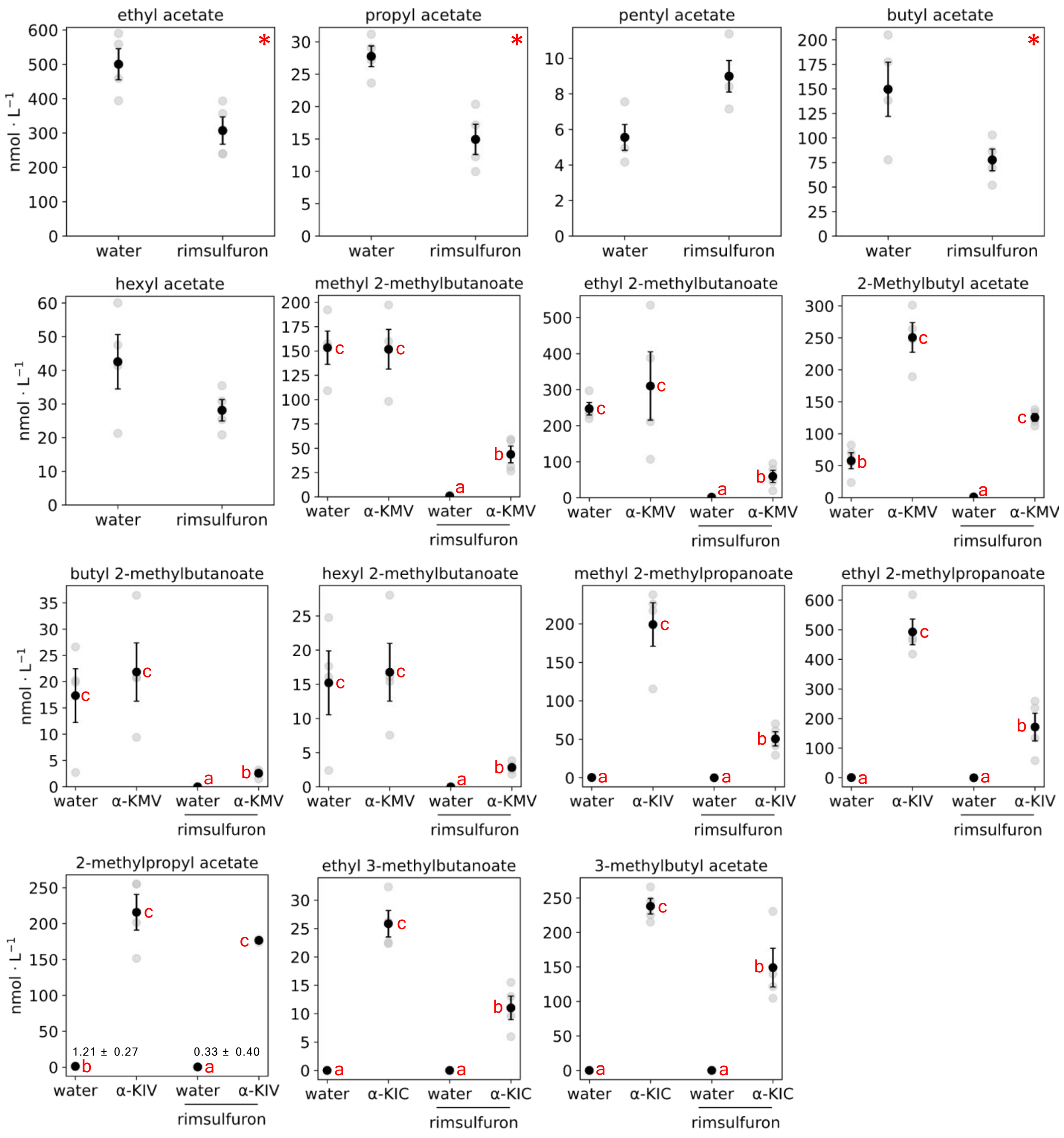

Supplementary Figure S4. Volatile headspace concentrations of 'Empire' apple fruit peels treated with water or rimsulfuron and fed branched-chain α-ketoacids. Presented as means ± SE of four biological reps. α-KMV = α-keto-β-methylvalerate; α-KIV = α-ketoisovalerate; α-KIC = α-ketoisocaproate. Significantly different straight-chain ester concentrations are denoted by * (two-tailed two-sample equal variance t-test, α=0.05). Significantly different branched-chain ester concentrations are denoted by different letters adjacent to means (data transformed for statistical analysis via log(x+1) due to unequal variance of α-ketoacid fed samples; Tukey's test, α=0.05). The concentrations of 2-methylpropyl acetate in tissues not fed with α-KIV are shown in figure.

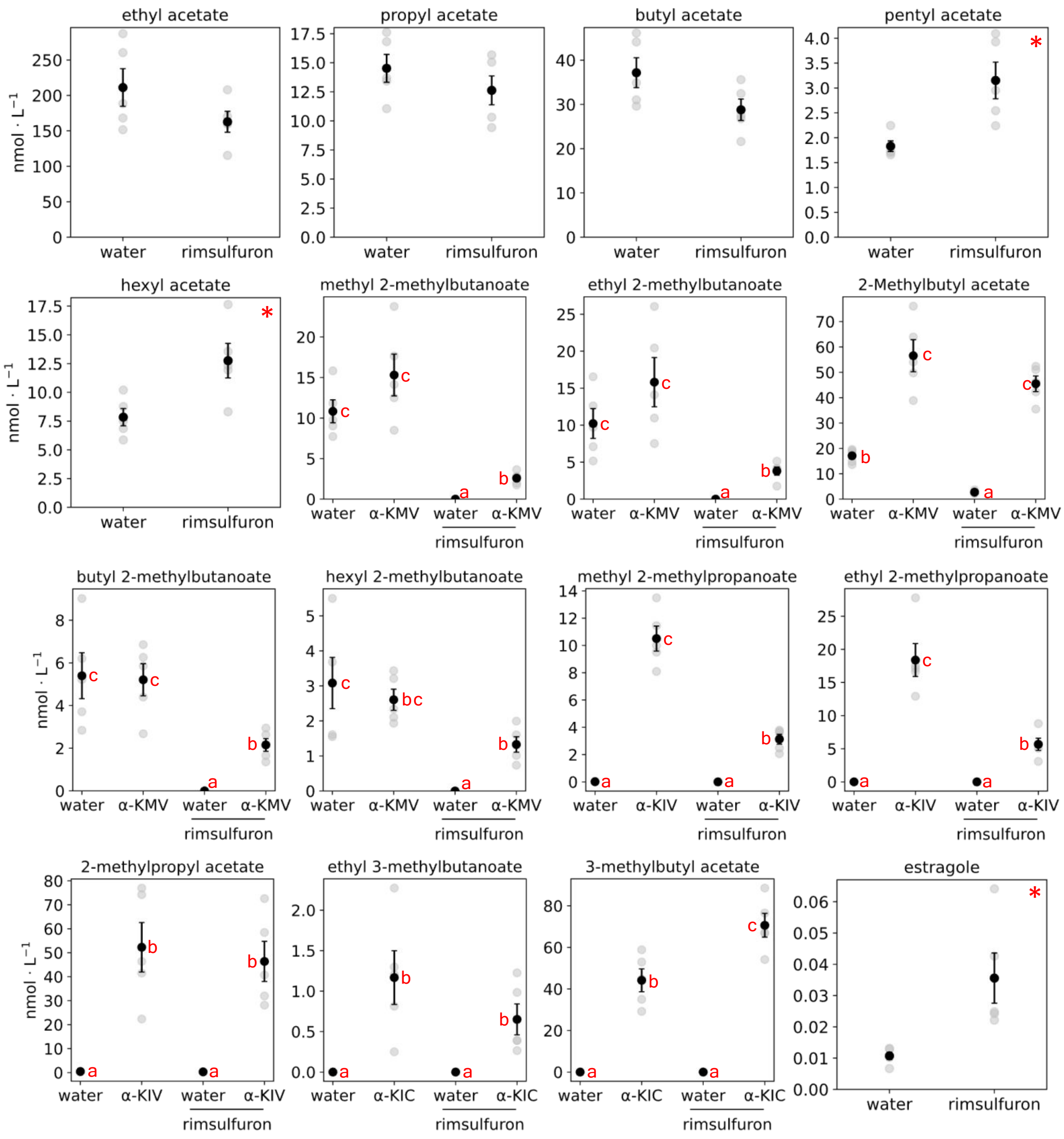

Supplementary Figure S5. Volatile headspace concentrations of ‘Jonagold’ apple fruit peels treated with water or rimsulfuron and fed branched-chain α-ketoacids. Presented as means ± SE of five biological reps. α-KMV = α-keto-β-methylvalerate; α-KIV = α-ketoisovalerate; α-KIC = α-ketoisocaproate. Significantly different straight-chain ester concentrations are denoted by * (two-tailed two-sample equal variance t-test, α=0.05). Significantly different branched-chain ester concentrations are denoted by different letters adjacent to means (data transformed for statistical analysis via log(x+1) due to unequal variance of α-ketoacid fed samples; Tukey’s test, α=0.05).

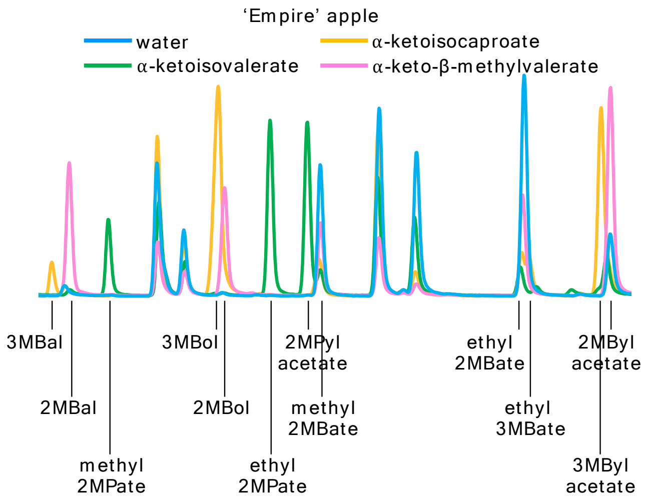

Supplemental Figure S6. Representative total ion chromatogram sections from incubation chamber headspace of ripening 'Empire' apple fruit peels fed with methanol and either water or a branched-chain α-ketoacid. 2MBal = 2-methylbutanal, 2MBate = 2-methylbutanoate, 2MBol = 2-methylbutanol, 2MByl = 2-methylbutyl, 2MPate = 2-methylpropanoate, 2MPyl = 2-methylpropyl, 3MBal = 3-methylbutanal, 3MBate = 3-methylbutanoate, 3MBol = 3-methylbutanol, 3MByl = 3-methylbutyl.

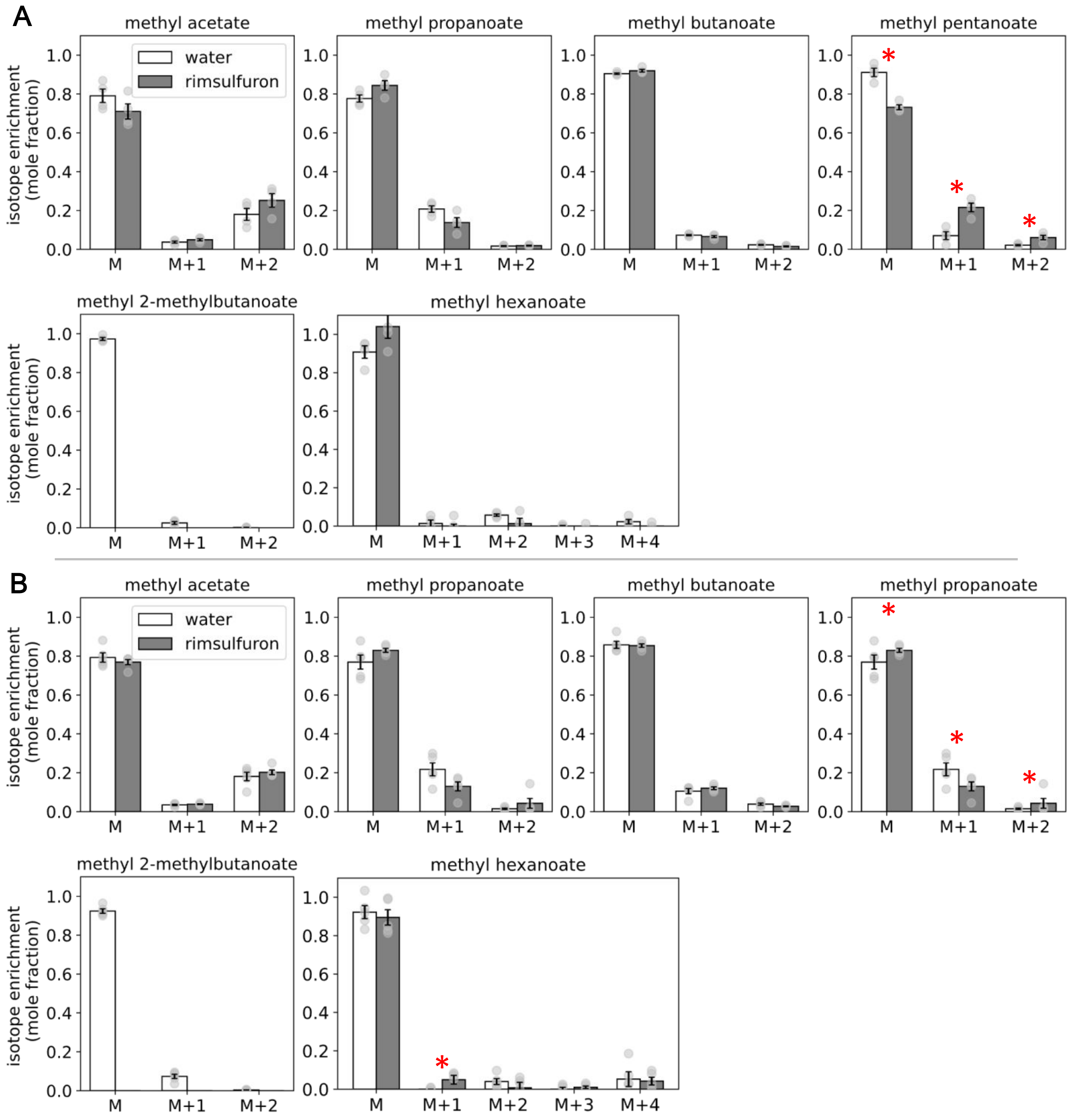

Supplemental Figure S7. Mass isotopolog distribution of methyl esters from ripening 'Empire' (A) and 'Jonagold' (B) apple fruit peels treated with water or rimsulfuron and fed 1,2-$^{13}C_2$ acetate and methanol. Presented as means ± SE of ≥ four biological reps. Significantly different distributions are denoted by * (two-tailed two-sample equal variance t-test, α=0.05). For more information on expected labeling patterns see Sugimoto et al., 2021[17].

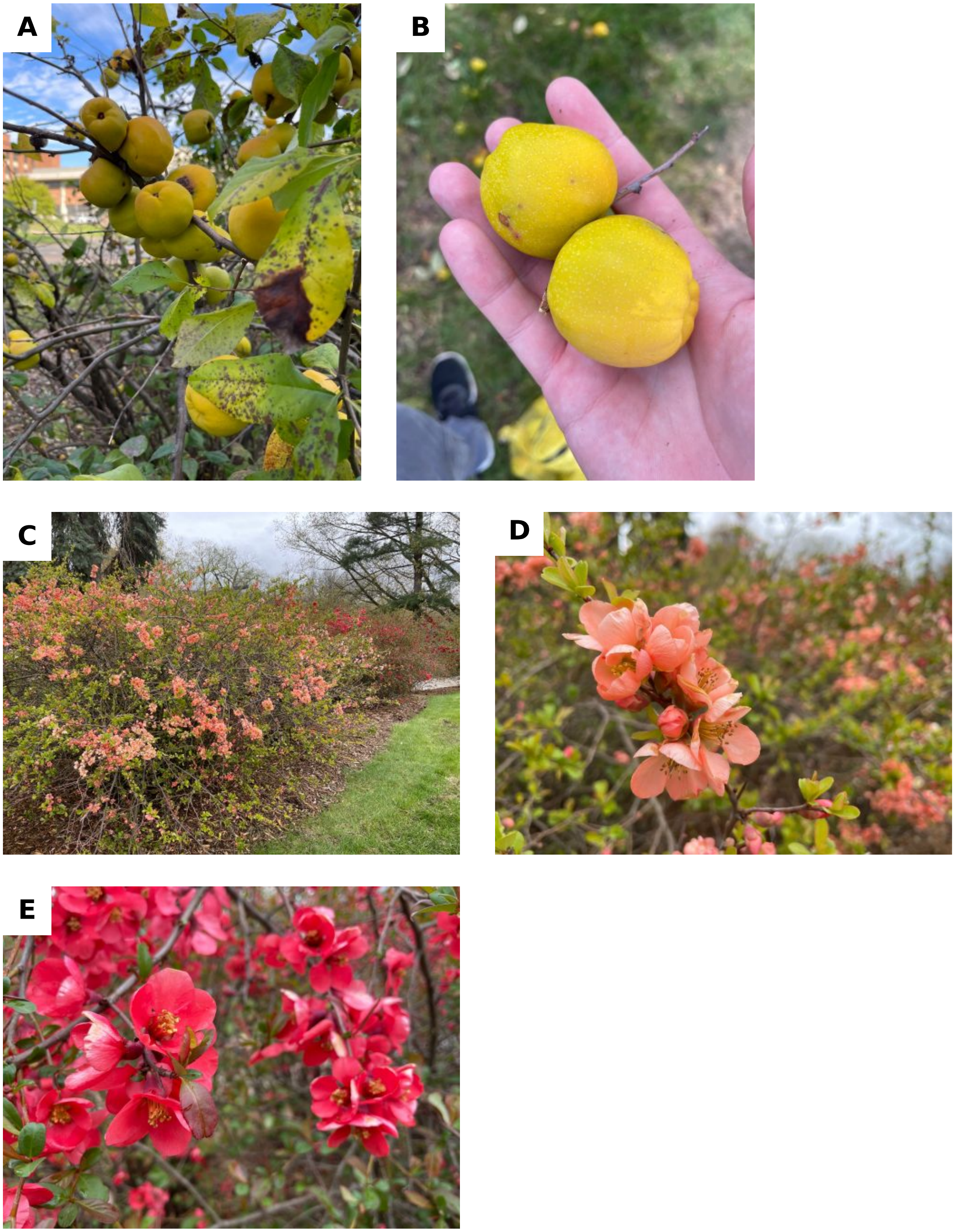

Supplementary Figure S8. Photos of flowering quince (*Chaenomeles ×superba*). A-B) 'Dr. Banks Pink' fruit on October 6th, 2022. C-E) Flowers of flowering quince on May 10th, 2023. Pinkish flowers = 'Dr. Banks Pink'; red flowers = 'Crimson Beauty' (not used in this study). Photo credit: Philip Engelgau.
